# The Effect of Calcium Source on Pb and Cu Remediation Using Enzyme-Induced Carbonate Precipitation

**DOI:** 10.3389/fbioe.2022.849631

**Published:** 2022-02-11

**Authors:** Lin Wang, Wen-Chieh Cheng, Zhong-Fei Xue

**Affiliations:** ^1^ School of Civil Engineering, Xi’an University of Architecture and Technology, Xi’an, China; ^2^ Shaanxi Key Laboratory of Geotechnical and Underground Space Engineering (XAUAT), Xi’an, China

**Keywords:** heavy metal, biomineralization, urease enzyme, urea hydrolysis, remediation efficiency

## Abstract

Heavy metal contamination not only causes threat to human health but also raises sustainable development concerns. The use of traditional methods to remediate heavy metal contamination is however time-consuming, and the remediation efficiency may not meet the requirements as expected. The present study conducted a series of test tube experiments to investigate the effect of calcium source on the lead and copper removals. In addition to the test tube experiments, numerical simulations were performed using Visual MINTEQ software package considering different degrees of urea hydrolysis derived from the experiments. The remediation efficiency degrades when NH_4_^+^ and OH^−^ concentrations are not sufficient to precipitate the majority of Pb^2+^ and Cu^2+^. It also degrades when CaO turns pH into highly alkaline conditions. The numerical simulations do not take the dissolution of precipitation into account and therefore overestimate the remediation efficiency when subjected to lower Pb(NO_3_)_2_ or Cu(NO_3_)_2_ concentrations. The findings highlight the potential of applying the enzyme-induced carbonate precipitation to lead and copper remediations.

## Introduction

Heavy metal contamination not only raises sustainable concerns but also causes threats to human health and plants (Kumari et al., 2016; Jiang et al., 2019; Rahman et al., 2020; Chen et al., 2021a; Tan et al., 2022). In the past few decades, remediation methods including replacement, soil flushing, electrokinetic remediation, chemical precipitation, ion exchange, and phytoremediation were developed (Mena et al., 2016; Kumari et al., 2017; Kumari et al., 2018; Chen and Achal, 2019; Duarte-Nass et al., 2020; Ahenkorah et al., 2021; Hu L. et al., 2021; Jiang et al., 2021). However, they are usually time-consuming and may cause secondary pollution (Achal et al., 2012; Nancharaiah et al., 2016; Kappaun et al., 2018; Xue et al., 2022; Yan et al., 2021; Duan et al., 2021a; Cheng et al., 2021; Duan et al., 2021b). Furthermore, heavy metal ions may be re-released into surrounding environments when subjected to the change in environmental conditions (Hu et al., 2021b; Bai et al., 2017; Bai et al., 2021; Xue et al., 2021). In light of this, a sustainable countermeasure that is able to immobilize heavy metal ions using an environmental-friendly manner is considered of great necessity. Enzyme-induced carbonate precipitation (EICP) is a novel biogeotechnical technique and has been widely applied to strengthen calcareous sands (Ahenkorah et al., 2021; Wei et al., 2021; Yuan et al., 2021; Chen et al., 2021b). Notwithstanding that, the application of the EICP method to the remediation of heavy metals is rarely reported. Urease enzyme can be derived from animals and plants. The principle of applying EICP technology to heavy metal remediation is to catalyze urea hydrolysis by the urease enzyme toward discharging ammonia and carbonate ions (NH_4_^+^ and CO_3_^2-^). By CO_3_^2-^ and Ca^2+^, carbonates are formed and precipitated with heavy metal ions (also referred to as immobilization of heavy metal) where Ca^2+^ ions are derived from the addition of calcium source (Mugwar and Harbottle, 2016). The biochemical reactions that count on urea hydrolysis are shown in Eqs (1–3) (Mobley and Hausinger, 1989; Bai et al., 2019; Bai et al., 2020).

$$CO{\left(NH_{2}\right)}_{2}+H_{2}O\to 2NH_{3}+CO_{2},$$ (1)

$$2NH_{3}+2H_{2}O↔2NH_{4}^{+}+2OH^{-},$$ (2)

$$CO_{2}+2OH^{-}↔HCO_{3}^{-}+OH^{-}↔CO_{3}^{2-}+H_{2}O.$$ (3)

In recent years, EICP technology is also developed as a remediation method for immobilizing inorganic contaminates. Gowthaman et al. (2021) used urease enzyme combined with bone meal to form calcium phosphate biocementation toward decreasing toxic gaseous ammonia by approximately 90%. Furthermore, Moghal et al. (2020) introduced the EICP method that incorporates citric acid (C_6_H_8_O_7_) and ethylene diamine tetra-acetic acid (EDTA) to study the desorption response of heavy metal ions to the soil. Although the literature immobilizes contaminants by EICP technology solely or by a combination of EICP technology with other additions, studies on the mechanisms affecting the remediation efficiency are remarkably limited (Achal and Mukherjee, 2015; Li et al., 2015; Kang and So, 2016). The objectives of this study are 1) to investigate the response of urease activity to high Pb^2+^ and Cu^2+^ concentrations by performing a series of test tube experiments, 2) to investigate the effect of calcium source on the speciation of carbonate precipitation, and 3) to reveal the mechanisms affecting the remediation efficiency for Pb and Cu removals.

## Materials and Methods

### Urease Extraction

*Canavalia ensiformis* is a known plant to be rich in urease enzyme (Yuan et al., 2020). In the present work, urease enzyme was extracted from *Canavalia ensiformis*. *Canavalia ensiformis* was first ground using a grinding machine and then sieved using a sieve with 150-μm mesh opening. The solution containing jack bean and 30% ethanol was centrifuged for 30 min and then stored at 4°C for 4 h. After that, the supernatant was second centrifuged for 1 h and then stored at −20°C for future use.

### Urease Activity Measurement

At a temperature of 30°C, 1 international unit (IU) of enzyme activity corresponds to the amount of 1.0 μmol catalyzed transformation in 1 min (Mobley and Hausinger, 1989). In this study, the amount of product (i.e., ammonium ion) catalyzed by urease was measured by Nessler’s reagent colorimetric method. Nessler’s reagent and potassium sodium tartrate were added, and the absorbance was measured by using a spectrophotometer after waiting for 10 min. The calibration line was set up by the same method prior to the measurement, and the measured absorbance was then substituted to the calibration line to get the concentration of ammonium ions. The urease activity being 342.7 U/g was therefore measured in the present work, which is classed as low activity.

### Evaluation of Remediation Efficiency

**FIGURE 1:**
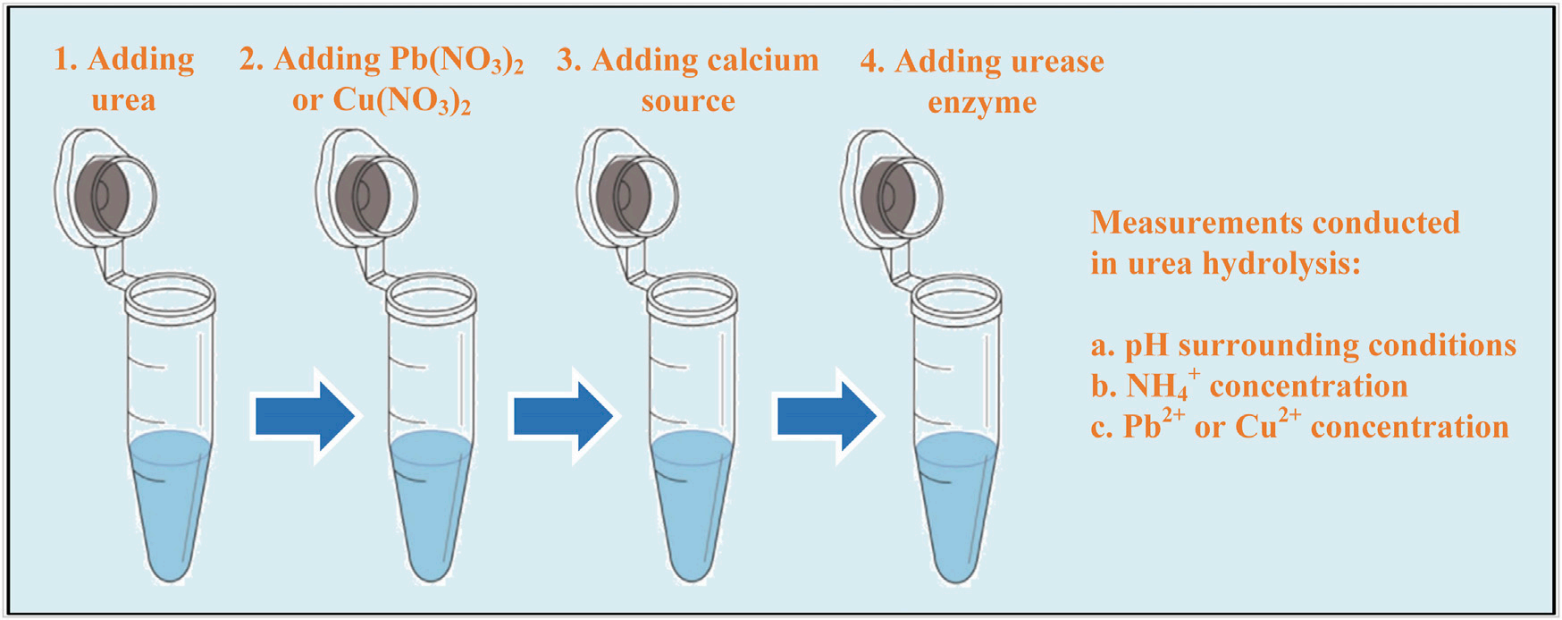
Schematic illustration of the flowchart applied to the test tube experiments.

To investigate the effect of calcium source on copper and lead remediations, a series of test tube experiments were conducted in this study. Three calcium sources, namely, calcium chloride (CaCl_2_), calcium acetate (Ca(CH_3_COO)_2_), and calcium oxide (CaO) were adopted in these test tube experiments to investigate heavy metal (lead nitrate (Pb(NO_3_)_2_) and copper nitrate (Cu(NO_3_)_2_)) remediations. A solution applied to the test tube experiments consisted of distilled water, urea, Pb(NO_3_)_2_ or Cu(NO_3_)_2_, calcium source, and urease enzyme. The concentration of calcium source used in the test tube experiments was 0.25 M, while the urea concentration was 0.5 M. This concentration range was set to produce enough carbonate to precipitate calcium ions. Heavy metal concentrations adopted included both the low and high values of 5 mM, 10 mM, 30 mM, 40 mM, and 50 mM, respectively. The preparation of the solution is demonstrated in Figure 1. Measurements of pH and NH_4_^+^ concentration, precipitation mass, and remaining heavy metal ion concentration were performed not only to evaluate the urease activity but also for the final assessment of the remediation efficiency. All of the test tube experiments have three replicates. An error bar has already been added to figures where necessary. Statistical analysis indicates that the coefficient of variance for the test tube experiments is below 10%, which is within the requirement being the coefficient of variance of 15% for usual, accessible experimental measurements.

### Numerical Simulation

In addition to the test tube experiments, numerical simulations were performed using Visual MINTEQ software package. The numerical simulations, in fact, refer to the concentration of ions (e.g., NH_4_^+^ and CO_3_^2-^) measured right after the test tube experiments, rather than referring to biochemical processes involved in catalyzing urea hydrolysis. In addition, their stoichiometric ratio sticks to 2:1 (Gat et al., 2017). The concentration of calcium source applied to the simulations was 0.25 M, while NH_4_^+^ concentrations (corresponding to the degree of urea hydrolysis) varying in a 10–200 mM range was adopted. The concentration of Pb^2+^ or Cu^2+^ fell in a 5–100 mM range. Given that the degree of urea hydrolysis may affect heavy metal removal, the change in NH_4_^+^ concentration benefits us to see how the speciation of carbonate precipitation varies with the degree of urea hydrolysis.

## Results and Discussion

### Urea Hydrolysis Response

**FIGURE 2:**
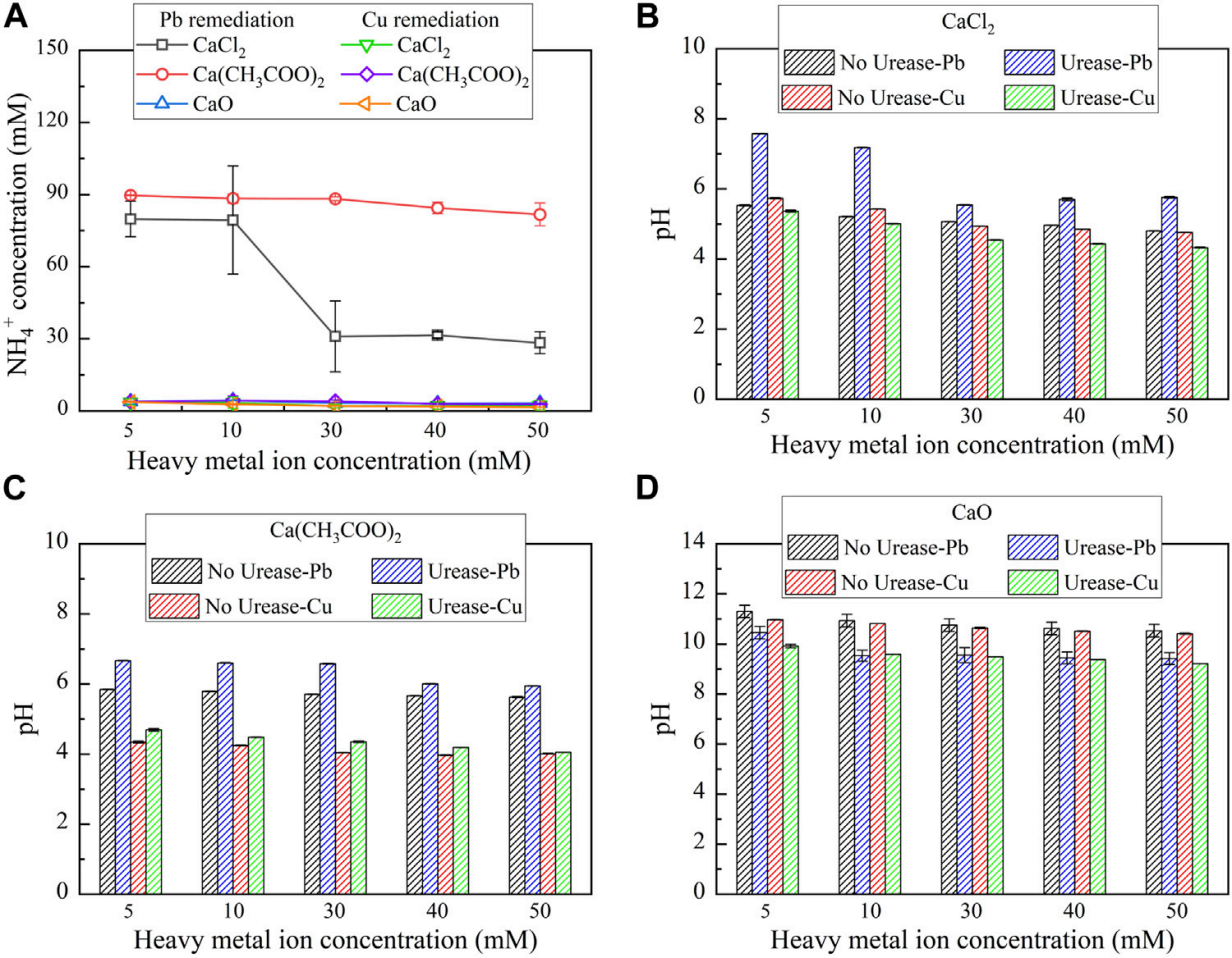
Ammonium ion concentration and pH vs heavy metal concentration: **(A)** NH_4_^+^ concentration, **(B)** CaCl_2_, **(C)** Ca(CH_3_COO)_2_, and **(D)** CaO.

Urease has been widely applied to catalyzing urea hydrolysis. Ammonium ions (NH_4_^+^) and hydroxide ions (OH^−^) are known to be discharged after urea hydrolysis (see Eqs 1–3), and therefore, NH_4_^+^ and pH are considered as the key indicators of describing the degree of urea hydrolysis (Fisher et al., 2017; Sun et al., 2021). In biomineralization, attending higher degrees of urea hydrolysis is considered of great necessity to improve heavy metal remediation efficiency. The relationships of NH_4_^+^ and pH vs heavy metal ion concentration under three calcium sources, CaCl_2_, Ca(CH_3_COO)_2_, and CaO, respectively, are shown in Figure 2. Under CaCl_2_, NH_4_^+^ concentration remaining high (approximately 70 mM) is noted when lower Pb(NO_3_)_2_ concentrations (i.e., 5-10 mM) are confronted (see Figure 2A). When subjected to higher Pb(NO_3_)_2_ concentrations (i.e., 30-50 mM), there appears a decline in NH_4_^+^ concentration. The higher the concentration of Pb^2+^, the more significant will be the effect on the urease activity and the lower will be the degree of urea hydrolysis. pH remaining in a 7–8 range may be appropriate in securing the urease activity toward elevating the degree of urea hydrolysis (see Figure 2B). The highest NH_4_^+^ concentration being about 105 mM is attained under Ca(CH_3_COO)_2_, corresponding to the highest degree of urea hydrolysis (see Figure 2C). The urease activity, associated with Ca(CH_3_COO)_2_, remains higher than that under CaCl_2_ when pH is falling in the 6–7 range. NH_4_^+^ concentration under CaO approaches 0, indicating the absence of urea hydrolysis. Provided there appears remediation efficiency, its attendance relies upon chemical precipitation that is not relevant to the “biomineralization” but to the reaction between Ca^2+^ and OH^−^ (Eq. 4).

$$Ca^{2+}+2OH^{-}\to Ca{\left(OH\right)}_{2}↓.$$ (4)

Despite that, the chemical precipitation could depress the urease activity by strongly alkaline pH of surrounding conditions, degrading the degree of urea hydrolysis (Mobley and Hausinger, 1989; Schock et al., 1995; Ferris et al., 2004; Hu et al., 2021c) (see Figure 2D). On the other hand, NH_4_^+^ concentration under any of the calcium sources always approaches 0 for Cu remediation (see Figure 2A). This is to say that the effect of Cu^2+^ on the urease activity is much higher than the effect of Pb^2+^, thereby reducing the degree of urea hydrolysis. To this end, chemical precipitation is supposed to be present here because of the absence of urea hydrolysis. pH higher than 9 is observed under CaO, while pH lower than 6 is noted under CaCl_2_ and Ca(CH_3_COO)_2_ (see Figures 2B–D). In short, the highest degree of urea hydrolysis presents under Ca(CH_3_COO)_2_, followed by CaCl_2_ and CaO, for the Pb remediation. However, the degree of urea hydrolysis is rather low under any of the calcium sources for the Cu remediation, indicating that there appears remediation efficiency, and chemical precipitation is present here.

### Effect of Calcium Source

**FIGURE 3:**
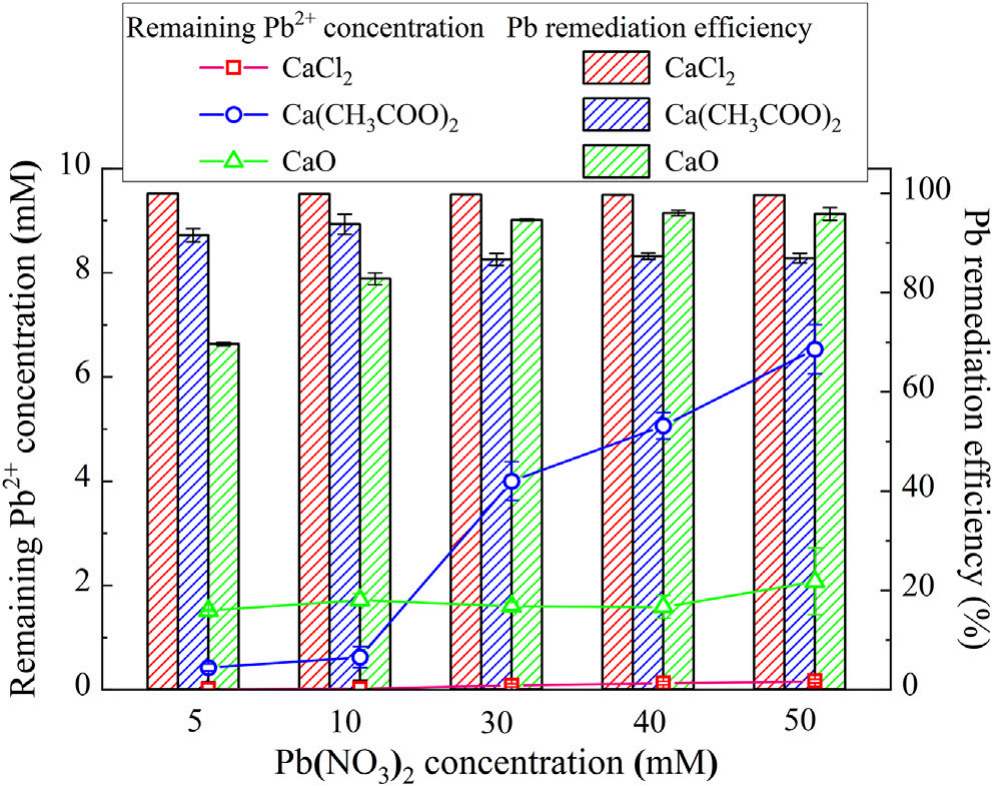
Relationships of the remaining Pb^2+^ concentration and the remediation vs Pb(NO_3_)_2_ concentration under CaCl_2_, Ca(CH_3_COO)_2_, and CaO, respectively.

**FIGURE 4:**
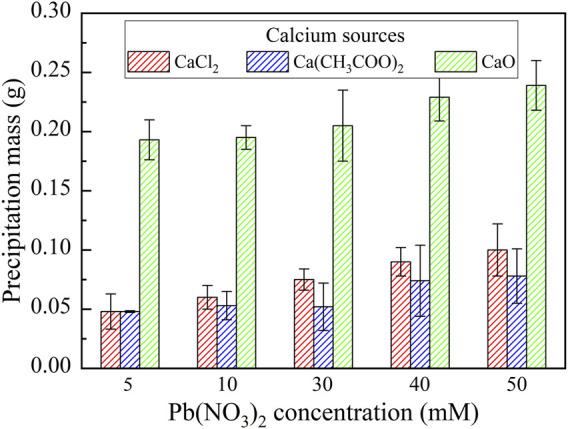
Relationships of the precipitation mass vs Pb(NO_3_)_2_ concentration under CaCl_2_, Ca(CH_3_COO)_2_, and CaO respectively.

This part primarily aims to link the effect of calcium source to the Pb remediation efficiency. The remediation efficiency is defined as follows:

$$Remediation\;efficiency\;=\left[\left(C_{I}-C_{R}\right)/C_{I}\right]\times 100\%,$$ (5)

where *C*_I_ represents the initial heavy metal concentration and *C*_R_ corresponds to the remaining heavy metal concentration. The lower the remaining heavy metal ion concentration, the higher will be the remediation efficiency. The relationships of the remaining Pb^2+^ concentration and the remediation efficiency vs Pb(NO_3_)_2_ concentration under CaCl_2_, Ca(CH_3_COO)_2_, and CaO, respectively, are shown in Figure 3. Under CaCl_2_, the remediation efficiency higher than 99% (the highest in the present work), with the remaining Pb^2+^ concentration way below 2 mM, is observed, although the precipitation mass is not the highest (see Figure 4). It can be inferred that given the second highest degree of urea hydrolysis, biotic precipitation may contribute to the attendance of the highest remediation efficiency. In addition to the biotic precipitation, the highest remediation efficiency could not be achieved without the participation of chemical precipitation. Considering that the chemical precipitation is present earlier than the biotic precipitation, the occupation of Pb^2+^ by the chemical precipitation first depresses the effect of Pb^2+^ on the urease activity, forming the biotic precipitation by catalyzing urea hydrolysis to assure the highest remediation efficiency.

Under Ca(CH_3_COO)_2_, the degree of urea hydrolysis is the highest, discharging more NH_4_^+^ and OH^−^. The remediation efficiency decreases with the increase in Pb(NO_3_)_2_ concentration. These results indicate that the chemical and biotic precipitations inhibit the effect of Pb^2+^ on the urease activity. However, the depression by the chemical and biotic precipitations turns out to be less significant when subjected to higher Pb(NO_3_)_2_ concentrations. It can also be observed from Figure 4 that the precipitation mass under Ca(CH_3_COO)_2_ can be compared to that under CaCl_2_ and is much below that under CaO. On the other hand, under CaO, the degree of urea hydrolysis is the lowest among the three calcium sources; therefore, urea hydrolysis and biotic precipitation that appear are not present here. For this reason, the chemical precipitation, Ca(OH)_2_, presents. Ca(OH)_2_ due to its higher solubility product then transfers to Pb(OH)_2_.

$$Pb^{2+}+2OH^{-}\to Pb{\left(OH\right)}_{2}↓.$$ (6)

As discussed, strongly alkaline conditions (higher than 10) could depress urease activity and also raise the dissolution potential of chemical precipitation (see Figure 2D). This addresses the degradation in the remediation efficiency when subjected to lower Pb(NO_3_)_2_ concentrations. The role of Pb^2+^ by the chemical precipitations is not only to reduce their effect on the urease activity but also to become more significant when subjected to higher Pb(NO_3_)_2_ concentrations. The remediation efficiency increases with the increase in Pb(NO_3_)_2_ concentration, with the highest measured at about 95%. On the whole, for the Pb remediation, the highest remediation efficiency is attained using CaCl_2_, whereas the highest precipitation mass is attained using CaO. Furthermore, the Pb remediation efficiency decreases and increases with the increasing Pb(NO_3_)_2_ concentration under Ca(CH_3_COO)_2_ and CaO, respectively.

**FIGURE 5:**
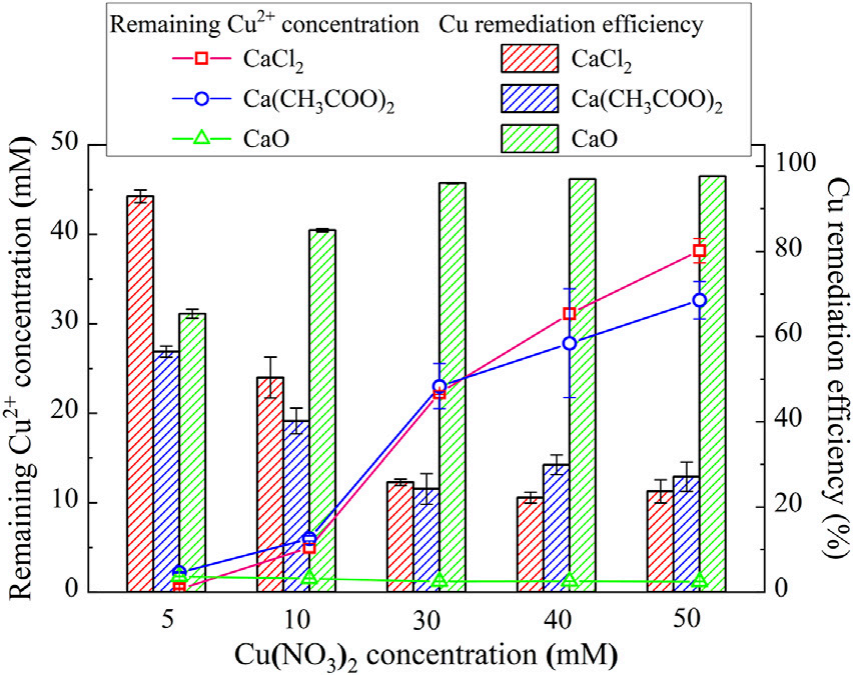
Relationships of the remaining Cu^2+^ concentration and the remediation vs Cu(NO_3_)_2_ concentration under CaCl_2_, Ca(CH_3_COO)_2_, and CaO, respectively.

**FIGURE 6:**
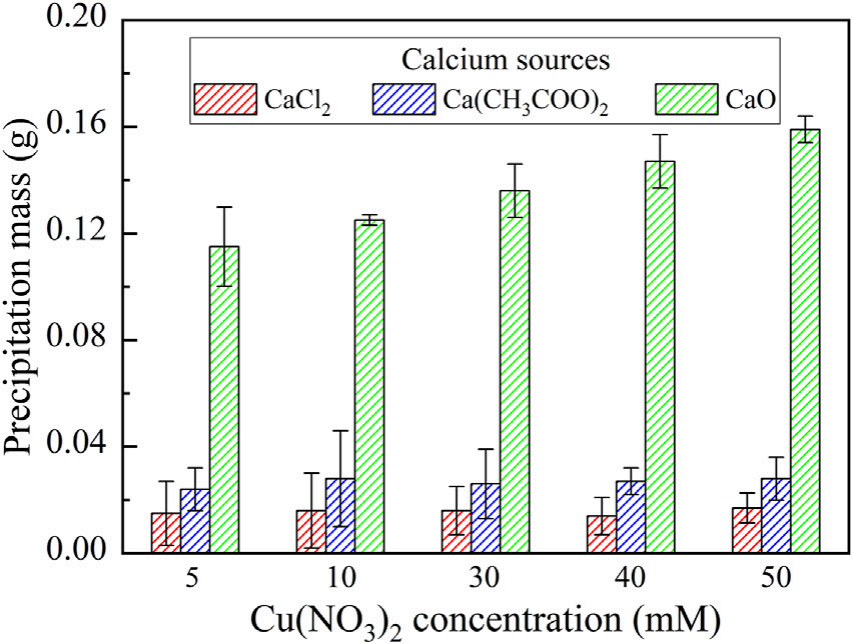
Relationships of the precipitation mass vs Cu(NO_3_)_2_ concentration under CaCl_2_, Ca(CH_3_COO)_2_, and CaO, respectively.

This part primarily targets to enhance our knowledge linkage between the effect of calcium source and Cu remediation. The relationships of the remaining Cu^2+^ concentration and the remediation efficiency vs Cu(NO_3_)_2_ concentration under CaCl_2_, Ca(CH_3_COO)_2_, and CaO, respectively are shown in Figure 5. NH_4_^+^ concentration is much below 15 mM under any of the calcium sources, which corresponds to a very low degree of urea hydrolysis (see Figure 2A). Despite that, the highest remediation efficiency is attained using CaO, followed by CaCl_2_ and Ca(CH_3_COO)_2_, most likely because of the formation of chemical precipitation (Shaw and Raval, 1961). The remediation efficiency beyond 90% is attained using CaCl_2_ when subjected to Cu(NO_3_)_2_ concentration at 5 mM, and it decreases with the increase in Cu(NO_3_)_2_ concentration. In contrast, under CaO, the remediation efficiency increases with the increasing Cu(NO_3_)_2_ concentration, with the maximum when subjected to Cu(NO_3_)_2_ concentration varying in a 30–50 mM range. On the other hand, when subjected to Cu(NO_3_)_2_ concentration at 5 mM, the remediation efficiency of up to 55% is attained using Ca(CH_3_COO)_2_, and it decreases with the increase in Cu(NO_3_)_2_ concentration. Similarly, the highest precipitation mass is still attained using CaO, followed by Ca(CH_3_COO)_2_ and CaCl_2_ (see Figure 6). On the whole, the effect of Cu^2+^ badly causes urease to lose its activity. This leads to the inability of catalyzing urea hydrolysis to form biotic precipitation. The highest remediation efficiency is attained using CaO. Considering the main cause leading to the difference in remediation efficiency under CaCl_2_, Ca(CH_3_COO)_2_, and CaO remains unclear, and it would be discussed later in this article.

### Effect of Precipitation Speciation

#### Pb Removal

**FIGURE 7:**
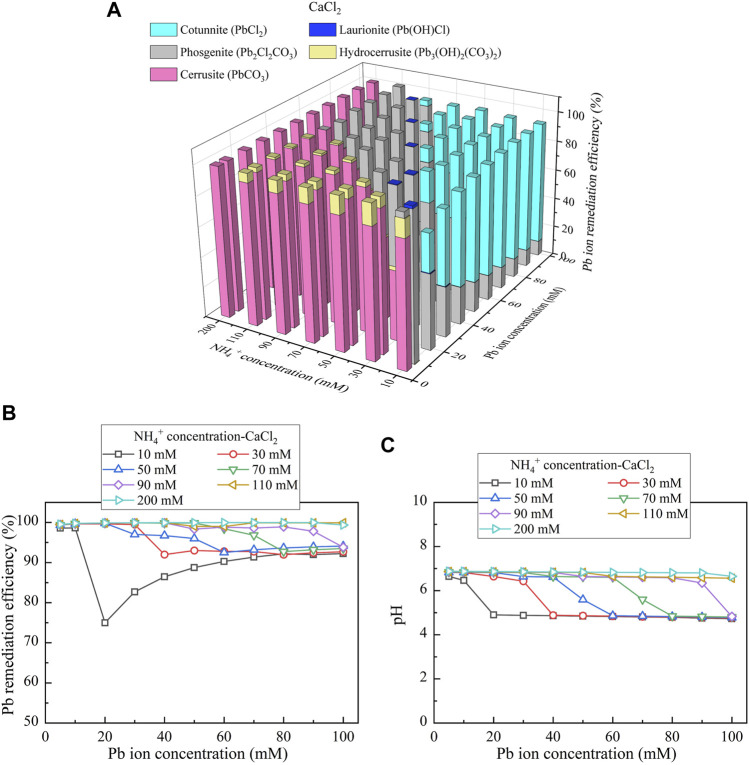
**(A)** Variations of the Pb remediation efficiency against different Pb^2+^ concentration ranges considering NH_4_^+^ concentrations varying in a 10–200 mM range, **(B)** variations of the Pb remediation efficiency against different Pb^2+^ concentration ranges, and **(C)** variations of pH of surrounding conditions against different Pb^2+^ concentration ranges (calcium source = CaCl_2_).

To reveal the speciation of precipitation, a series of simulations were conducted using Visual MINTEQ software package considering different degrees of urea hydrolysis (i.e., NH_4_^+^ concentration) derived from the test tube experiments. The three calcium sources applied to the experiments also participated in the simulations. The simulated results would give the speciation of precipitation and also benefit to revealing the mechanisms affecting the remediation efficiency. Considering CaCl_2_ as the calcium source, the relationships of the remediation efficiency vs NH_4_^+^ concentration and Pb(NO_3_)_2_ concentration are depicted in Figure 7A. There are five speciations, including cotunnite (PbCl_2_), laurionite (Pb(OH)Cl), phosgenite (Pb_2_Cl_2_CO_3_), hydrocerussite (Pb_3_(OH)_2_(CO_3_)_2_), and cerussite (PbCO_3_). Considering the discharge of CO_3_^2-^ and NH_4_^+^ only presents when there appears urea hydrolysis, associated precipitation is also referred to as the “biotic” precipitation. In contrast, precipitation not relevant to CO_3_^2-^ is termed as the “chemical” precipitation. To this end, the later three precipitations are categorized as the biotic precipitations, whereas the first two are classified as the chemical precipitations. PbCO_3_, Pb_3_(OH)_2_(CO_3_)_2_, and Pb_2_Cl_2_CO_3_ present when the degree of urea hydrolysis is high enough to precipitate the majority of Pb^2+^. In total, 1 mM CO_3_^2-^ for PbCO_3_ is needed to thoroughly precipitate 1 mM Pb^2+^, whereas for Pb_3_(OH)_2_(CO_3_)_2_, 2 mM CO_3_^2-^ is required to precipitate 3 mM Pb^2+^. When the degree of urea hydrolysis is low and cannot discharge enough CO_3_^2-^ and NH_4_^+^, Pb(OH)Cl and PbCl_2_ present. The chemical precipitation in some cases may not be as stable as the biotic precipitation because of its higher solubility product toward causing some difficulty in capsulizing heavy metal ions and raising the concern of their migration. This would eventually degrade the remediation efficiency (Edwards et al., 1992; Baltpurvins et al., 1996).

Figures 7B,C respectively show the variations in the remediation efficiency and pH against different Pb^2+^ concentration ranges considering NH_4_^+^ concentrations (i.e., degree of urea hydrolysis) varying in a 10–200 mM range. The remediation efficiency of approximately 100% (corresponding to 100 mM Pb^2+^) is attained using 200 mM NH_4_^+^ (see Figure 7B). In contrast, there appears a substantial reduction in the remediation efficiency to 75% (corresponding to 15 mM Pb^2+^) when Pb^2+^ and NH_4_^+^ concentrations are 20 and 10 mM, respectively. In total, 10 mM NH_4_^+^ corresponds to 5 mM CO_3_^2-^, and 5 mM CO_3_^2-^ can only precipitate 10 mM Pb^2+^, in accordance with the speciation of Pb_2_Cl_2_CO_3_ (see Figure 7A). The remaining 5 mM Pb^2+^ is therefore precipitated by the chemical precipitation (i.e., PbCl_2_). The degradation in the remediation efficiency is due to the lack of CO_3_^2-^ and NH_4_^+^. There appears the other reduction to 90% (corresponding to 36 mM Pb^2+^) when Pb^2+^ and NH_4_^+^ concentrations are 40 and 30 mM, respectively. In total, 30 mM NH_4_^+^ corresponds to 15 mM CO_3_^2-^. As a result, 15 mM CO_3_^2-^ can only precipitate 30 mM Pb^2+^, producing the biotic precipitation (i.e., Pb_2_Cl_2_CO_3_) (see Figure 7A). The chemical precipitation (i.e., PbCl_2_) is to precipitate the remaining 6 mM Pb^2+^. It is noteworthy that the degradations in the remediation efficiency take place when pH of surrounding conditions become below 6 (see Figure 7C). These results indicate that improving the remediation efficiency relies not only on the degree of urea hydrolysis but also on the pH of surrounding conditions (Krajewska, 2016). pH falling in a 6–9 range causes urease to possess hydrolytic activity (Krajewska, 2016). These also satisfactorily address the reduction in the degree of urea hydrolysis when subjected to Pb^2+^ concentrations higher than 30 mM and pH below 6 (see Figures 2A,B).

**FIGURE 8:**
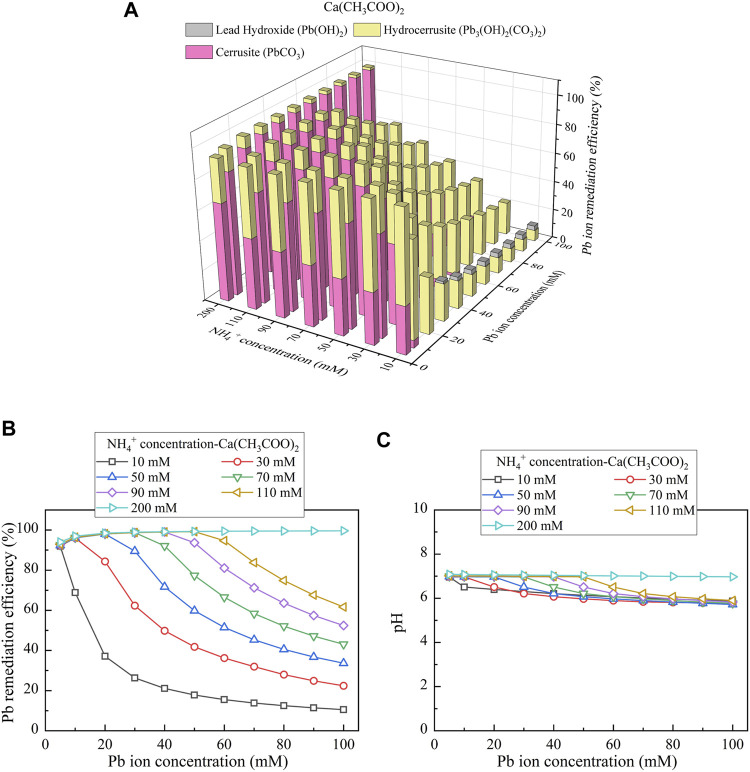
**(A)** Variations of the Pb remediation efficiency against different Pb^2+^ concentration ranges considering NH_4_^+^ concentrations varying in a 10–200 mM range, **(B)** variations of the Pb remediation efficiency against different Pb^2+^ concentration ranges, and **(C)** variations of pH of surrounding conditions against different Pb^2+^ concentration ranges (calcium source = Ca(CH_3_COO)_2_).

Considering Ca(CH_3_COO)_2_ as the calcium source, the relationships of the remediation efficiency vs NH_4_^+^ concentration and Pb(NO_3_)_2_ concentration are shown in Figure 8A. There are three speciations of precipitation, including cerussite (PbCO_3_), hydrocerussite (Pb_3_(OH)_2_(CO_3_)_2_), and lead hydroxide (Pb(OH)_2_), where the last is classified as the chemical precipitation, and the other two are classified as the biotic precipitation. The remediation efficiency of approximately 100% (corresponding to 100 mM Pb^2+^) is attained via 200 mM NH_4_^+^ (see Figure 8B). Similarly, there appears a reduction in the remediation efficiency when CO_3_^2-^ and NH_4_^+^ concentrations are not high enough. The degradations in the remediation efficiency initiate when pH stays away from 7 (see Figure 8C). They develop further when pH drops consistently.

**FIGURE 9:**
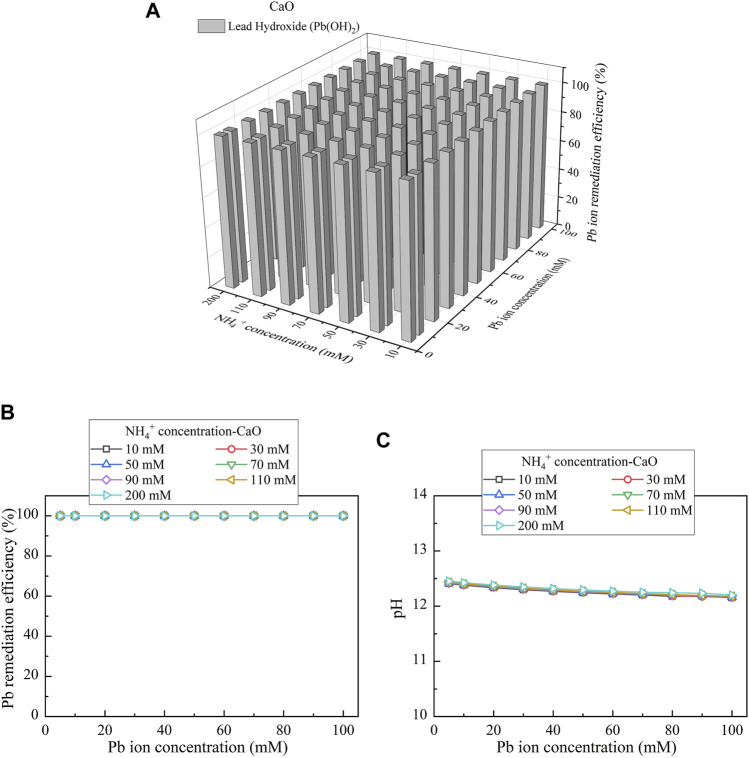
**(A)** Variations of the Pb remediation efficiency against different Pb^2+^ concentration ranges considering NH_4_^+^ concentrations varying in a 10–200 mM range, **(B)** variations of the Pb remediation efficiency against different Pb^2+^ concentration ranges, and **(C)** variations of pH of surrounding conditions against different Pb^2+^ concentration ranges (calcium source = CaO).

When CaO is considered as the calcium source, the relationships of the remediation efficiency vs NH_4_^+^ concentration and Pb(NO_3_)_2_ concentration are shown in Figure 9A. The precipitation corresponds to one single speciation, namely, Pb(OH)_2_. Pb(OH)_2_ is categorized as the chemical precipitation. The remediation efficiency as high as 100% is attained irrespective of NH_4_^+^ concentration, indicating the absence of urea hydrolysis (see Figure 9B). As discussed, Ca(OH)_2_ precipitates earlier than Pb(OH)_2_, and the former turns pH into strongly alkaline conditions, causing the dissolution of precipitation (see Figure 9C). The dissolution of precipitation, in turn, degrades the remediation efficiency when subjected to lower Pb(NO_3_)_2_ concentrations (see Figure 3). pH turns however into weakly alkaline conditions when subjected to higher Pb(NO_3_)_2_ concentrations. This tackles difficulty in catalyzing urea hydrolysis under strongly alkaline conditions and therefore raises the remediation efficiency. The experimental results appear to conflict with the simulated ones, most likely due to the neglection of precipitation dissolution by the simulations. The remediation efficiency remaining very high under pH above 12 provides testimony of the aforesaid argument (see Figure 9C).

#### Cu Removal

**FIGURE 10:**
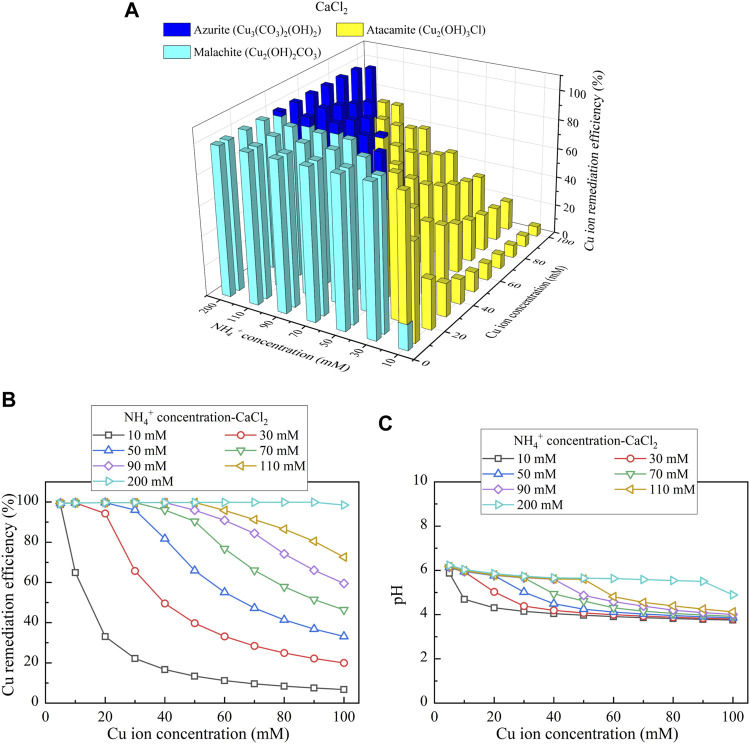
**(A)** Variations of the Cu remediation efficiency against different Cu^2+^ concentration ranges considering NH_4_^+^ concentrations varying in a 10–200 mM range, **(B)** variations of the Cu remediation efficiency against different Cu^2+^ concentration ranges, and **(C)** variations of pH of surrounding conditions against different Cu^2+^ concentration ranges (calcium source = CaCl_2_).

**FIGURE 11:**
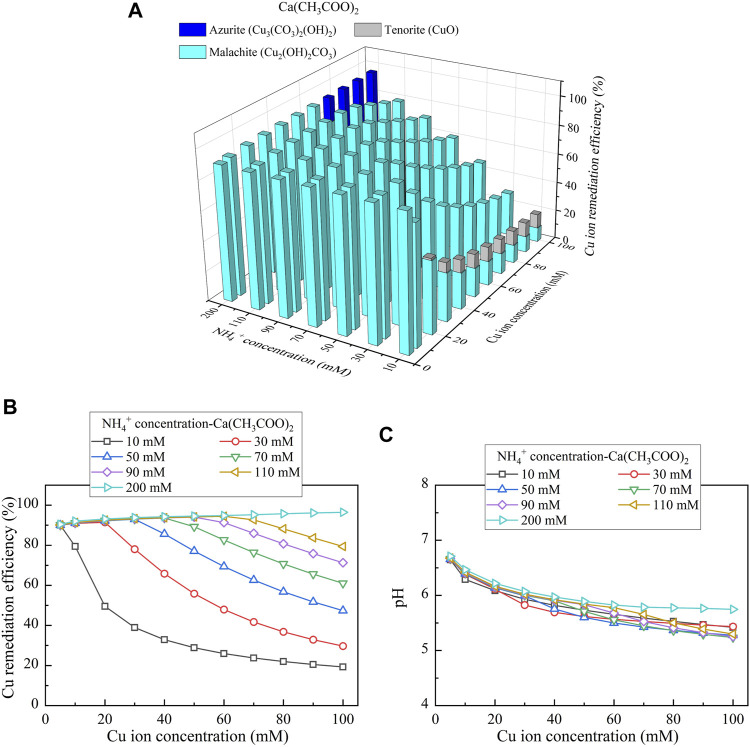
**(A)** Variations of the Cu remediation efficiency against different Cu^2+^ concentration ranges considering NH_4_^+^ concentrations varying in a 10–200 mM range, **(B)** variations of the Cu remediation efficiency against different Cu^2+^ concentration ranges, and **(C)** variations of pH of surrounding conditions against different Cu^2+^ concentration ranges (calcium source = Ca(CH_3_COO)_2_).

**FIGURE 12:**
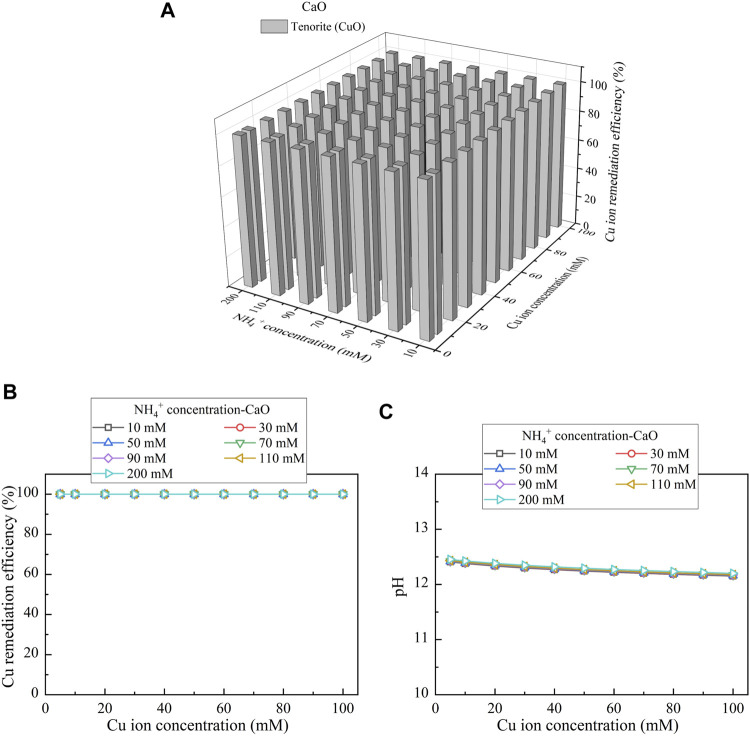
**(A)** Variations of the Cu remediation efficiency against different Cu^2+^ concentration ranges considering NH_4_^+^ concentrations varying in a 10–200 mM range, **(B)** variations of the Cu remediation efficiency against different Cu^2+^ concentration ranges, and **(C)** variations of pH of surrounding conditions against different Cu^2+^ concentration ranges (calcium source = CaO).

This part presents the simulated results applied to the Cu removal. Considering CaCl_2_ as the calcium source, the relationships of the Cu remediation efficiency vs NH_4_^+^ concentration and Cu(NO_3_)_2_ concentration are shown in Figure 10A. Three speciations of precipitation are identified. Azurite (Cu_3_(CO_3_)_2_(OH)_2_) and malachite (Cu_2_(OH)_2_CO_3_) are classified as the biotic precipitations, while atacamite (Cu_2_(OH)_3_Cl) is classified as the chemical precipitation. Furthermore, under Ca(CH_3_COO)_2_, Cu_3_(CO_3_)_2_(OH)_2_, tenorite (CuO), and Cu_2_(OH)_2_CO_3_ are recognized, as shown in Figure 11A. Moreover, using CaO as calcium source, single one speciation, namely, CuO, is identified, as shown in Figure 12A. It can be seen from Figures 10B,C, 11B,C that under CaCl_2_ and Ca(CH_3_COO)_2_, the lack of CO_3_^2-^ and NH_4_^+^ triggers a degradation in the remediation efficiency. In addition to the lack of CO_3_^2-^ and NH_4_^+^, the degradation in the remediation efficiency is also counted on the remediation efficiency. Under CaO, it dissolves in water to form Ca(OH)_2_, and Ca(OH)_2_ turns pH into highly alkaline conditions toward affecting the urease activity and eventually degrading the remediation efficiency (see Figures 12B,C). The degradation in the remediation efficiency is however absent here as the numerical simulation neglects the precipitation dissolution. To summarize, the effect of Cu^2+^ can more significantly reduce urease activity. The highest remediation efficiency is attained using CaO. Such high remediation efficiency still presents when subjected to lower Cu(NO_3_)_2_ concentrations, which is not in line with the experimental results. The neglection of precipitation dissolution by the simulations is deemed as the main cause leading to the discrepancy. The remediation efficiency degrades when CO_3_^2-^ and NH_4_^+^ concentrations are not high enough to precipitate the majority of Cu^2+^. In addition, the remediation efficiency also degrades when pH turns into highly alkaline conditions, induced by the addition of CaO.

### Mechanisms Affecting Remediation Efficiency

**FIGURE 13:**
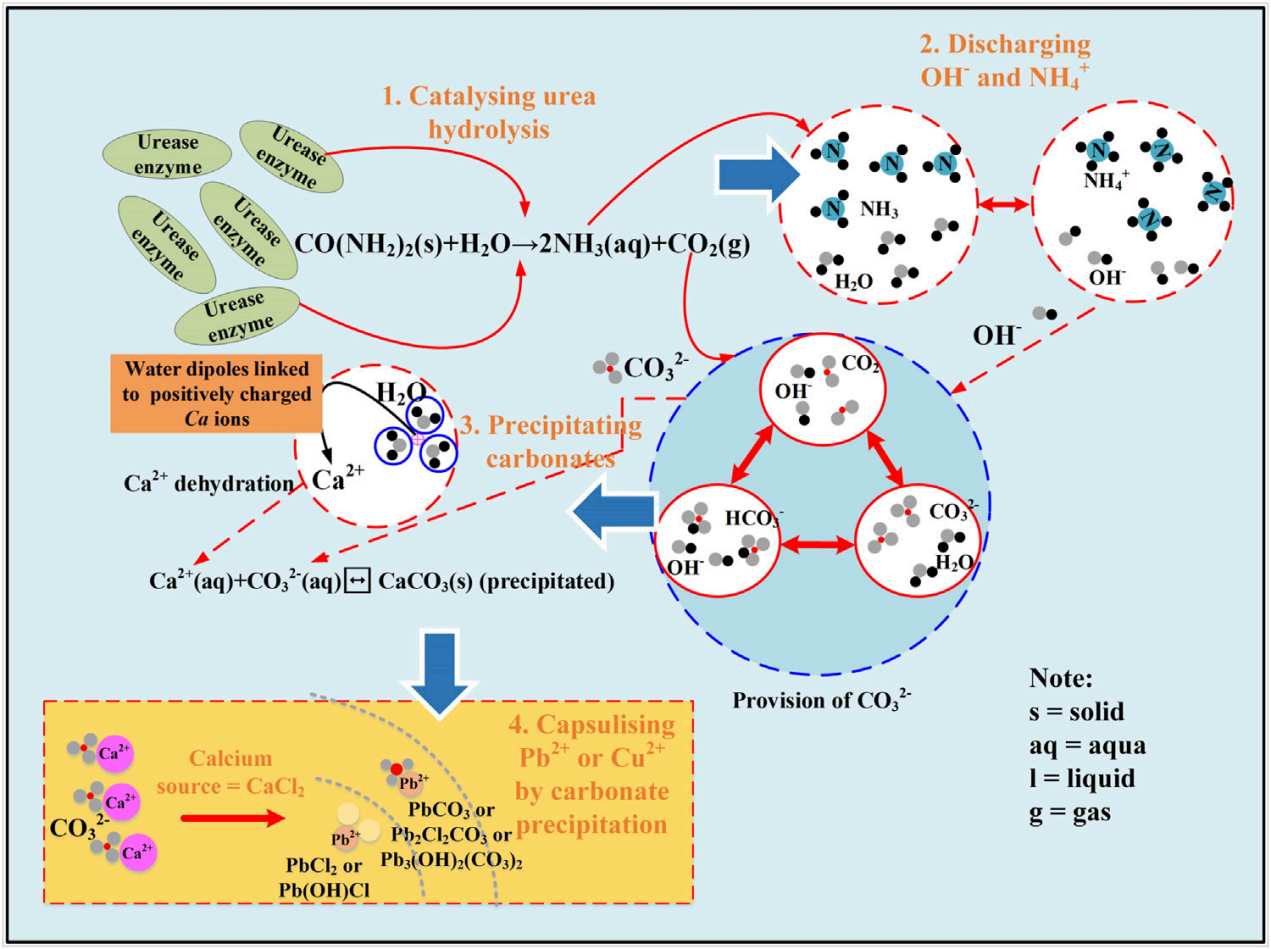
Schematic illustration of the mechanisms affecting the remediation efficiency.

Heavy metal ions can significantly affect the urease activity and cause some difficulty in catalyzing urea hydrolysis and capsulizing heavy metal ions toward degrading the remediation efficiency. Urease can be capsulized by the addition of calcium sources, preventing loss of their activity. Under CaCl_2_ and Ca(CH_3_COO)_2_, NH_4_^+^ concentration for the Pb remediation remains as a high provide testimony of the aforesaid argument. As a result, the biotic precipitation can precipitate the majority of Pb^2+^ toward securing the remediation efficiency. However, under CaO, NH_4_^+^ concentration is approximately 5 mM, which also indicates the absence of urea hydrolysis. In this case, the remediation efficiency relies upon the chemical precipitation. The degradation in the remediation efficiency presents when subjected to lower Pb(NO_3_)_2_ concentrations. Ca(OH)_2_ turning pH into highly alkaline conditions is considered as the leading cause resulting in the degradation of the remediation efficiency. Therefore, the degradation in the remediation efficiency not only relies on the provision of NH_4_^+^ and OH^−^ but also on the change in pH, induced by the addition of calcium source. NH_4_^+^ concentration for the Cu remediation is measured as low as approximately 5 mM, irrespective of calcium source. As a result, the remediation efficiency is attained not only by the biotic precipitation but also by the chemical precipitation. It is worth noting that under CaO, the reduction as appeared when subjected to lower Cu(NO_3_)_2_ concentrations in the test tube experiments is absent in the simulated results. The discrepancy is mainly attributed to the neglection of precipitation dissolution under highly alkaline conditions. To conclude, ensuring the degree of urea hydrolysis is deemed of great necessity when applying the enzyme-induced carbonate precipitation technology to heavy metal remediation. There are two leading causes affecting the remediation efficiency: one is the provision of NH_4_^+^ and OH^−^, and the other is the change in pH, resulting from the addition of calcium source (see Figure 13). Here, NH_4_^+^ and OH^−^ concentrations high enough can precipitate the majority of Pb^2+^ and Cu^2+^, corresponding to the biotic precipitation. When NH_4_^+^ and OH^−^ concentrations are not high enough to precipitate the majority of Pb^2+^ and Cu^2+^, the remediation efficiency is attained through a combination of biotic and chemical precipitations. On the other hand, CaO dissolves in water to produce Ca(OH)_2_ toward turning pH into highly alkaline conditions. Such alkaline conditions could not only depress the urease activity but also cause dissolution of the carbonate precipitation, thereby degrading the remediation efficiency. The degradation is absent in the simulated results because the simulations do not take the dissolution of carbonate precipitation into account.

## Conclusion

The test tube experiments were conducted to reproduce catalyzing urea hydrolysis using urease enzyme. The experimental and numerical results were applied to investigate the effect of calcium source on Pb and Cu removals. Based on the results and discussions, some main conclusions can be drawn as follows:

1) The remediation efficiency degrades when NH_4_^+^ and OH^−^ concentrations are not sufficient to precipitate the majority of Pb^2+^ and Cu^2+^. The remediation efficiency due to the lack of Pb^2+^ and Cu^2+^ is attained through a combination of the biotic and chemical precipitations.
2) Highly alkaline conditions could not only affect the urease activity but also cause dissolution of carbonate precipitation. CaO dissolves in water to produce Ca(OH)_2_, turning pH more alkaline, and degrading the remediation efficiency.
3) The numerical simulations are deemed useful in enhancing our knowledge linkage between the speciation of carbonate precipitation and the remediation efficiency and preventing unfavorable pH of surrounding conditions from affecting the degree of urea hydrolysis. Notwithstanding that, the simulations do not take the dissolution of precipitation into account and therefore overestimate the remediation efficiency when subjected to lower Pb(NO_3_)_2_ or Cu(NO_3_)_2_ concentrations.

## Data Availability

The original contributions presented in the study are included in the article/Supplementary Material, further inquiries can be directed to the corresponding author.
